# A transcriptomic atlas of macrophage polarization induced by inflammatory and reparative cytokines reveals cytokine-specific and synergistic polarization programs

**DOI:** 10.3389/fimmu.2026.1815547

**Published:** 2026-10-07

**Authors:** Gregory R. Lum, Bria M. Gorman, Silvana A. Sidhom, Oscar A. Davalos, Deepa K. Murugesh, Nicole F. Leon, Margarita V. Rangel, Nicholas R. Hum, Aimy Sebastian

**Affiliations:** Lawrence Livermore National Laboratory, Physical and Life Sciences Directorate, Livermore, CA, United States

**Keywords:** cytokine-mediated responses, gene expression profiling, inflammation and tissue repair, macrophage activation, macrophage plasticity

## Abstract

Macrophages are highly plastic immune cells derived from bone marrow progenitors that respond dynamically to environmental cues and coordinate inflammation, debris clearance, tissue remodeling, wound repair, and regeneration. Although traditionally classified into pro-inflammatory (M1) and anti-inflammatory (M2) phenotypes, macrophage activation is now recognized as a continuum of overlapping states shaped by complex signaling environments. However, how diverse microenvironmental cues shape macrophage states and functions remains incompletely defined. Here, we performed a systematic transcriptomic analysis of murine bone marrow-derived macrophages stimulated with a broad panel of inflammatory, regulatory, and reparative cytokines, both individually and in selected combinations. Our analysis identified distinct and overlapping transcriptional programs associated with classical, alternative, and hybrid activation states, and showed that combinatorial cytokine signals modulate these programs beyond the effects of individual stimuli. Notably, combining IL-4 or IL-13 with IL-6 or IL-33 enhanced gene-expression programs associated with tissue repair, angiogenesis, and metabolic reprogramming. Cytokine array analysis further supported distinct, combination-specific secretory responses. Addition of IL-33 to the IL-4 + IL-13 backbone increased secretion of the alternative activation-associated chemokines CCL17 and CCL22, whereas addition of IL-6 uniquely increased secretion of the immunoregulatory cytokine IL-10. The combinations also showed condition-dependent increases in inflammatory mediators, including TNF-α, IL-18, CXCL1, and, to a lesser extent, IL-1β. Together, these findings support distinct mixed secretory programs rather than a uniform macrophage activation state. Overall, this study provides a systematic framework for understanding how individual and combinatorial cytokine cues shape macrophage transcriptional programs. This comprehensive dataset serves as a resource for interpreting macrophage heterogeneity in complex inflammatory and tissue-repair microenvironments and for prioritizing candidate regulatory pathways for future mechanistic investigation.

## 1 Introduction

Macrophages are highly plastic immune cells that adopt diverse functional states in response to local microenvironmental cues. In tissues, they contribute to host defense, maintain homeostasis, and coordinate responses to infection, injury, and inflammation (1). Their innate ability to assume distinct inflammatory, regulatory, and reparative phenotypes enables rapid adaptation to changing pathological conditions (2). This plasticity is particularly important following tissue injury, when macrophages integrate signals from cytokines, growth factors, and stromal cells to coordinate the transition from acute inflammation to resolution and tissue repair (2, 3).

Traditionally, macrophages have been classified into a binary framework: classically activated (M1) macrophages, which are pro-inflammatory and microbicidal, or alternatively activated (M2) macrophages, which act to damp down inflammation, promote immune tolerance, and support tissue repair and remodeling (4). However, accumulating evidence demonstrates that macrophage activation exists along a fluid continuum, with cells frequently exhibiting overlapping or hybrid features depending on the precise combinations of stimuli they encounter (2, 5). This multidimensional spectrum complicates efforts to categorize macrophage identity within complex tissue microenvironments, such as sites of sterile injury, chronic inflammation, or active wound healing, where diverse biochemical and cellular cues collectively shape functional phenotypes.

To account for this functional heterogeneity, the alternative activation spectrum has been expanded into specialized operational subtypes (M2a, M2b, M2c, and M2d), each induced by distinct microenvironmental stimuli and associated with specialized biological programs (6). M2a macrophages are generally associated with tissue repair and matrix deposition; M2b cells with immunoregulatory activity; M2c macrophages with tissue remodeling and resolution; and M2d cells with angiogenesis and, in certain contexts, tumor progression (7–9). Although this classification highlights the diversity of macrophage activation states, these operational categories have often been studied under highly constrained, single-cytokine conditions *in vitro*. Consequently, systematic comparative analyses of macrophages exposed to diverse individual and combinatorial cytokine environments remain limited (10–12).

This gap is particularly relevant in complex tissue microenvironments, such as sites of acute tissue injury and resolving inflammation, which contain a rich and dynamic cytokine milieu that shapes macrophage behavior in highly context-dependent ways. Within these microenvironments, individual cytokines drive distinct functional programs: IL-4 and IL-13 are canonical drivers of M2a-like reparative states in both human and murine macrophages (13, 14), while IL-33 promotes alternative activation via the IL-33/ST2 axis and may amplify IL-4- and IL-13-induced macrophage activation (14, 15). IL-6 can function both as a pro-inflammatory mediator and as an enhancer of IL-4-dependent pathways in macrophages through cooperative STAT3-STAT6 signaling, a mechanism highly conserved between human and murine macrophages (16–18). IL-10, a central anti-inflammatory cytokine, drives immunoregulatory macrophage programs and suppresses excessive inflammation through STAT3-dependent pathways (19), while TGF-β1 promotes M2-like phenotypes during tissue repair (20, 21). Although these cytokines trigger distinct transcriptional regulators, including STAT6, STAT3, IRF4, KLF4, and SMADs (19, 22, 23), how alternative activation-associated macrophage programs are shaped, reinforced, or redirected by combinations of these cytokines remains poorly understood.

To address this question, we performed controlled, side-by-side transcriptomic profiling of murine bone marrow-derived macrophages (BMDMs) exposed to thirteen individual and combinatorial cytokine regimens. We centered the analysis on IL-4- and IL-13-induced alternative activation-associated programs, evaluating their individual effects and their modification by IL-6 or IL-33. These responses were compared with IL-10- and TGF-β1-treated immunoregulatory and repair-associated conditions. IL-1β and IFNγ were included as inflammatory reference stimuli (24–26), whereas GM-CSF served as a distinct macrophage differentiation comparator relevant to inflammatory tissue environments (27–29). Together, these conditions enabled comparison of diverse cytokine-responsive macrophage states. Integrated gene-expression and pathway analyses identified distinct inflammatory, immunoregulatory, alternative activation-associated, metabolic, and tissue-remodeling features, which were complemented by multiplex cytokine analysis, nitrite measurements, and macrophage morphology. The cytokine array showed condition-specific secretory responses, with IL-33 addition to IL-4 + IL-13 increasing CCL17 and CCL22 secretion and IL-6 addition uniquely increasing IL-10 secretion. This study provides a transcriptomic and functional resource for interpreting macrophage plasticity and prioritizing candidate pathways for future investigation in inflammation resolution and tissue repair.

## 2 Methods

### 2.1 Animals

All animal experiments were approved by the Lawrence Livermore National Laboratory Institutional Animal Care and Use Committee. Wildtype C57BL/6 female mice aged 12–20 weeks were obtained from the Jackson Laboratory and used for all experiments.

### 2.2 Cell culture

Mice were euthanized by CO_2_ narcosis and secondary cervical dislocation. Mouse bone-marrow-derived mononuclear cells were isolated from tibias and femurs of mice as previously described (30). After processing to single cell suspension, 6×10^6^ bone marrow cells were plated on a 10 cm TC-untreated petri dish in 10 mL DMEM/F12 + 10% FBS supplemented with 100 ng/mL M-CSF (Thermo Fisher, cat# AF-315-02-50UG). These cells were differentiated into bone marrow-derived macrophages (BMDMs) for 7 days *in vitro*, with a half media change on day 1 and a full media change on day 5 of culture. BMDMs were reseeded in 12-well TC treated plates in 2mL DMEM/F12 + 10% FBS and stimulated for 24 hours (h) with 10 ng/mL IFNγ (Thermo Fisher, cat#AF-315-05-100UG), 10 ng/mL GM-CSF (Thermo Fisher, cat# 315-03-20UG), 10 ng/mL IL-1ß (Thermo Fisher, cat#211-11B-10UG), 10 ng/mL IL-4 (Thermo Fisher, cat# 214-14-20UG), 10 ng/mL IL-6 (Thermo Fisher, cat# 216-16-10UG), 10 ng/mL IL-10 (Thermo Fisher, cat# 210-10-10UG), 10 ng/mL IL-13 (Thermo Fisher, cat# 210-13-10UG), 10 ng/mL IL-33 (Thermo Fisher, cat# 210-33-10UG), 10 ng/mL TGF-β1 (Fisher Scientific, cat#7666MB005CF), 10 ng/mL IL-4 + 10 ng/mL IL-13, 10 ng/mL IL-4 + 10 ng/mL IL-13 + 10 ng/mL IL-33, or 10 ng/mL IL-4 + 10 ng/mL IL-6 + 10 ng/mL IL-13. For the GM-CSF-alone condition (Gmcsf_alone), bone marrow cells were cultured for 7 days in medium containing 100 ng/mL GM-CSF instead of M-CSF and were not exposed to M-CSF at any stage. M-CSF was used as the baseline differentiation condition for most experiments as it is a standard macrophage differentiation approach for comparative polarization studies, whereas GM-CSF was included as a representative inflammatory/mixed differentiation condition.

### 2.3 Cell imaging

BMDMs (1.5×10^5^ cells per well in a 24-well plate) were visualized at 20x magnification using phase-contrast microscopy (Agilent BioTek Citation C10).

### 2.4 Bulk RNA sequencing

Cultures of mouse BMDMs (n≥4) as prepared above were lysed in RLT buffer containing 1% β-mercaptoethanol. RNA was isolated using the RNeasy mini spin columns (Qiagen). Sequencing libraries were prepared using the Illumina Stranded mRNA Prep kit (Illumina) following manufacturer’s protocols and sequencing was performed using Illumina NextSeq 2000.

### 2.5 Bulk RNA sequencing data analysis

Sequencing quality was assessed using FastQC (31). Reads were aligned to the mouse reference genome (mm10) using STAR, and gene-level counts were generated with the featureCounts function from the Rsubread package (32, 33). Differentially expressed genes (DEGs) were identified using edgeR (34). To minimize batch effects, each cytokine-stimulated condition was compared with its corresponding untreated M-CSF-differentiated BMDM control from the same batch. Genes with a false discovery rate (FDR) < 0.05 and an absolute fold change ≥ 2 or log2 fold change (|log_2_FC|) ≥ 1 were considered significantly differentially expressed. Data visualization and Pearson correlation analysis were performed using custom R scripts. Similarity was defined as the Pearson correlation coefficient of log2 fold changes relative to naïve controls. Heatmaps were visualized using unscaled log₂ fold change values in the R package pheatmap, utilizing a symmetric color scale capped from -4 to 4. Rows were ordered via hierarchical clustering based on Euclidean distance. For correlation analyses and heatmaps, fold-change values relative to batch-matched controls were used, as combining expression values across batches can lead to misleading results. Principal component analysis (PCA) was performed in R. Functional enrichment analyses were conducted with clusterProfiler (35) and g:Profiler (36). Transcription-factor activity was inferred using decoupleR based on moderated t-statistics obtained with limma following voom normalization (37, 38). Sequence data have been deposited in the Gene Expression Omnibus (GEO) at the National Center for Biotechnology Information (NCBI) under accession number GSE315641.

### 2.6 Cytokine array analysis

Following 24 h stimulation under the indicated cytokine conditions, BMDM culture supernatants were collected, clarified by centrifugation, and stored at −80 °C until analysis. Cytokine concentrations were determined using the LEGENDplex mouse macrophage panel with v-bottom plate (BioLegend). Sample preparation and acquisition were performed according to manufacturer’s protocols. Samples were acquired using a Cytek Aurora flow cytometer. Analysis was performed within the LegendPlex analysis software v10 (BioLegend).

### 2.7 Nitrite quantification

To evaluate functional nitric oxide production across polarization conditions, RAW 264.7 murine macrophages were utilized as a highly standardized, responsive cell-line proxy, bypassing the high batch-to-batch variability and low baseline nitric oxide yield typically observed in primary BMDMs under non-classical activation conditions. Cells were cultured in DMEM + 10% FBS + 1% Pen/Strep and then seeded 1.5×10^5^ cells per well in a 24-well plate under standard culturing conditions. Nitrite accumulation in cell culture supernatants was used as an indirect measure of nitric oxide production. Following a 24 h stimulation with various cytokines, cell culture supernatants were collected and clarified for analysis. Nitrite concentration was determined using the Measure-iT High-Sensitivity Nitrite Assay Kit (Invitrogen). Sample preparation and acquisition were performed following manufacturer’s protocols. Fluorescence was read at 365/450nm excitation/emission using a Modulus Microplate Reader II. Nitrite concentrations were interpolated following the manufacturer’s suggested sodium nitrite standard curve preparation using cell culture media. Values were background corrected against the cell culture media.

## 3 Results

### 3.1 Differential gene regulation across cytokine polarization conditions

After 7 days of M-CSF-mediated differentiation, BMDMs were polarized for 24 h with the cytokines listed in **Table 1**, either individually or in combination, except for the Gmcsf_alone condition. For this condition, bone marrow cells were cultured with GM-CSF from day 0 and were not exposed to M-CSF at any stage. Gene expression profiles were generated by bulk RNA sequencing (**Figure 1A**; **Supplementary Table S1**).

**Table 1:** Experimental conditions.

Sample Name	Differentiation	Polarization
Mcsf	M-CSF	Control
Gmcsf_alone	GM-CSF	GM-CSF throughout
Mcsf.Gmcsf	M-CSF	GM-CSF
Mcsf.Ifng	M-CSF	IFNγ
Mcsf.IL1b	M-CSF	IL-1β
Mcsf.IL4	M-CSF	IL-4
Mcsf.IL6	M-CSF	IL-6
Mcsf.IL10	M-CSF	IL-10
Mcsf.IL13	M-CSF	IL-13
Mcsf.IL33	M-CSF	IL-33
Mcsf.IL4_IL13	M-CSF	IL-4 + IL-13
Mcsf.IL4_IL13_IL33	M-CSF	IL-4 + IL-13 + IL-33
Mcsf.IL4_IL13_IL6	M-CSF	IL-4 + IL-13 + IL-6
Mcsf.Tgfb	M-CSF	TGF-β1

GM-CSF, granulocyte-macrophage colony-stimulating factor; M-CSF, macrophage colony-stimulating factor.

**Figure 1:**
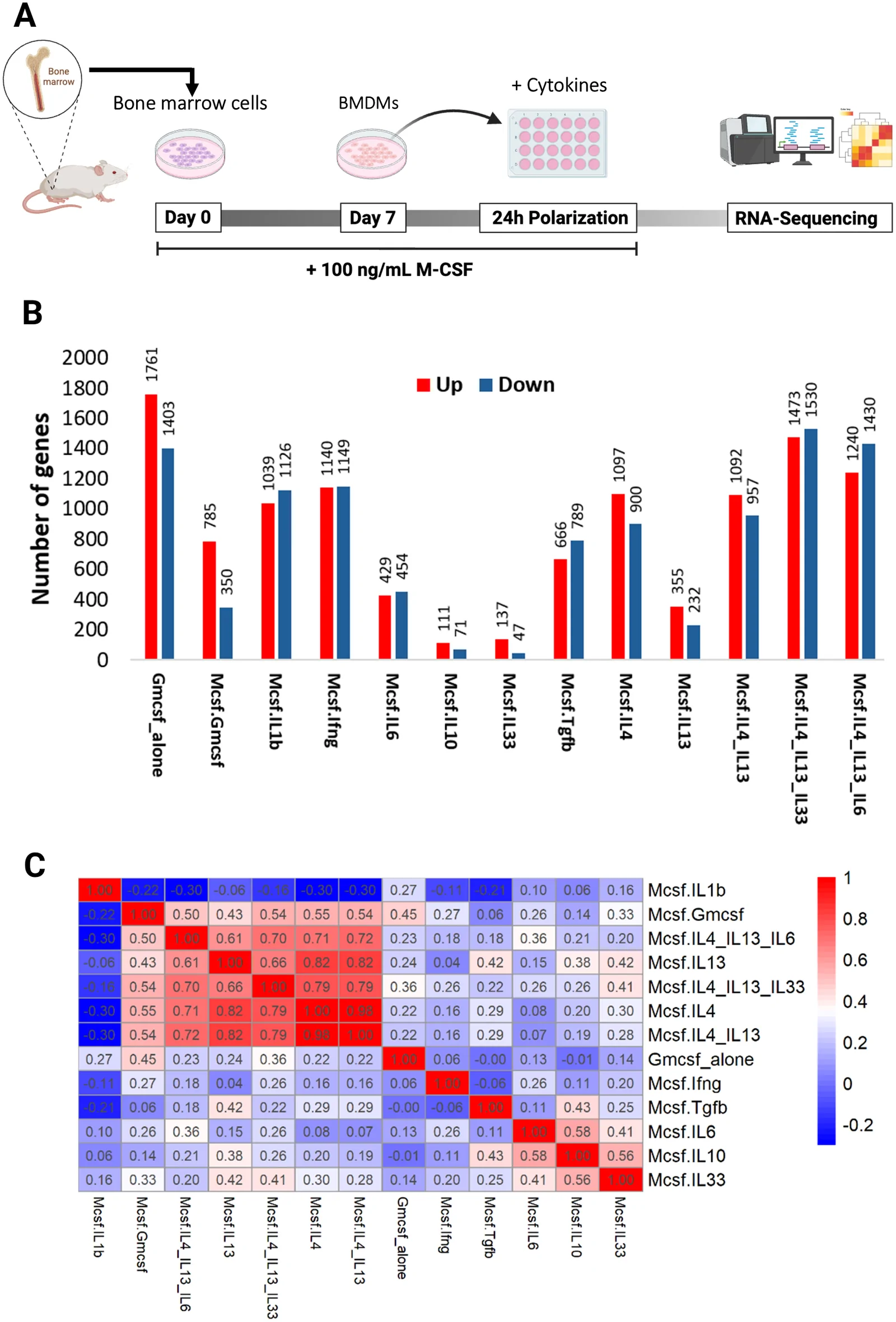
Transcriptomic profiling of cytokine-polarized mouse bone-marrow derived macrophages. **(A)** Graphical representation of the experimental workflow. Bone marrow cells isolated from C57BL/6 mice were treated with M-CSF for 7 days and polarized with selected cytokines for 24 h. Macrophages were then subjected to RNA sequencing. **(B)** Bar graph of total number of up- and down-regulated genes for each treatment as defined by FDR < 0.05 and log2FC ≥ 1 (upregulated) and log2FC ≤ -1 (downregulated). **(C)** Correlation plot comparing gene expression changes across all polarization conditions. Each treatment group is compared to every other group, with the diagonal representing self-comparisons (correlation coefficient = 1.0).

For each cytokine-polarized group, gene expression was compared with that of untreated M-CSF-differentiated BMDMs. The number of differentially expressed genes varied substantially across conditions (**Figure 1B**; **Supplementary Table S1**). Gmcsf_alone produced the largest transcriptional response, with the highest number of significantly upregulated and downregulated genes, indicating substantial transcriptional reprogramming during GM-CSF-mediated differentiation. In contrast, single-cytokine treatments of M-CSF-derived macrophages with IL-10, IL-33, IL-13, or IL-6 showed markedly fewer up- or down-regulated genes, suggesting a more restricted transcriptional response.

Combination treatments involving IL-4 and IL-13, as well as their inclusion with IL-33 or IL-6, two cytokines shown to amplify IL-4/IL-13 responses, produced a pronounced increase in the number of significantly regulated genes (**Figure 1B**; **Supplementary Table S1**). A correlation analysis revealed two major clusters of polarization states. The first cluster consisted of macrophages treated with IL-6 (Mcsf.IL6), IL-10 (Mcsf.IL10), and IL-33 (Mcsf.IL33), which exhibited related gene expression profiles. IL-6-treated macrophages exhibited moderate correlation with IL-10-treated macrophages (Pearson correlation r = 0.58), while IL-33-treated macrophages showed a similar correlation with IL-10-treated macrophages (r = 0.56) (**Figure 1C**). The TGF-β1-treated condition also clustered near the IL-10-treated condition, indicating partial similarity among these cytokine-induced transcriptional programs (**Figure 1C**).

The second cluster was primarily composed of those treated with alternative activation-associated cytokines IL-4, IL-13 or its combinations (Mcsf.IL4_IL13_IL6, Mcsf.IL13, Mcsf.IL4_IL13_IL33, Mcsf.IL4, Mcsf.IL4_IL13), reflecting a distinct transcriptional program characteristic of alternative (M2-like) activation (**Figure 1C**). M-CSF-derived macrophages treated with IL-4 were highly correlated with IL-4 + IL-13-treated macrophages (Pearson correlation r = 0.98), whereas IL-13-treated macrophages correlated less strongly with the IL-4 + IL-13 condition (r = 0.82). These results suggest that IL-4 and IL-13 drive largely overlapping gene expression programs, with IL-4 serving as the dominant transcriptional driver. The addition of IL-6 or IL-33 was associated with further changes in the IL-4 + IL-13 transcriptional response (**Supplementary Table S1**; **Figure 1B**).

The Mcsf.Gmcsf condition displayed moderate similarity (Pearson r > 0.50) to several states along the IL-4 axis. In contrast, Gmcsf_alone had low similarity to most other conditions, indicating that BMDMs differentiated with GM-CSF alone develop a distinct transcriptional program compared to those exposed to M-CSF (**Figure 1C**). Similarly, IFNγ- and IL-1β-treated macrophages exhibited divergent expression profiles with limited correlation to the other conditions (**Figure 1C**).

### 3.2 Hybrid activation states revealed by macrophage marker expression

To evaluate cytokine-associated macrophage programs, we analyzed selected genes commonly associated with inflammatory and alternative activation-related states, including M1, M2a, M2b, M2c, and M2d (**Figure 2A**). As expected, IFNγ treatment strongly induced classical inflammatory markers, including *Cxcl10*, *H2-Ab1*, and *Tnf*, consistent with an M1-like transcriptional program (**Figure 2A**; **Supplementary Table S1**) (26, 39). Consistent with this profile, IFNγ-treated RAW 264.7 macrophages showed a strong increase in nitrite production, a commonly used functional readout of iNOS-associated inflammatory macrophage activation (**Figure 2B**) (40).

**Figure 2:**
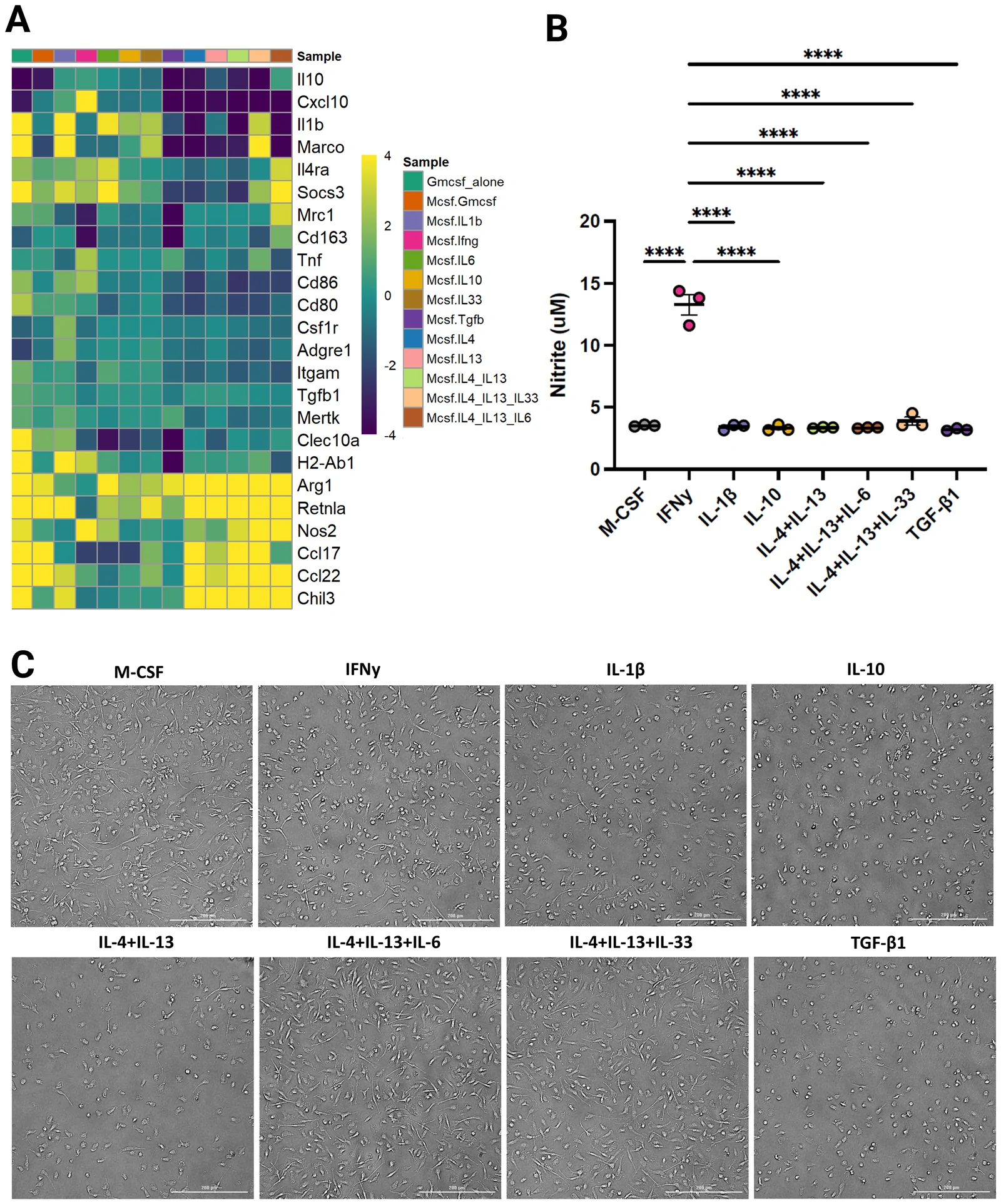
Cytokine cues induce distinct transcriptional and functional macrophage responses. **(A)** Heatmap of selected genes commonly associated with inflammatory and alternative activation-associated states. **(B)** Nitrite accumulation in conditioned media from RAW 264.7 cells after 24 h of each cytokine treatment regimen. (ordinary one-way ANOVA with Tukey’s multiple comparisons test; ****adjusted p < 0.0001). **(C)** Panel of cell images displaying cellular morphology for each of the cytokine treatment regimens in BMDMs. Scale bar: 200 μm.

The expression of anti-inflammatory cytokine *Il10* was significantly downregulated across most conditions, including GM-CSF, TGF-β1, and most IL-4-based combinations. A notable exception was the IL-4 + IL-13 + IL-6 group, which showed mild but significant upregulation, whereas a few other treatments showed no significant change (**Figure 2A**; **Supplementary Table S1**). This broad repression of *Il10* suggests that many cytokine cues, even those not canonically inflammatory, can shift macrophages away from an immunoregulatory state.

The pro-inflammatory cytokine *Il1b* was significantly upregulated in response to IL-1β, IL-6, GM-CSF, IL-10, IL-33, and notably also in the IL-4 + IL-13 + IL-33 combination, indicating that these diverse stimuli activate strong inflammatory programs, albeit through distinct signaling pathways (**Figure 2A**; **Supplementary Table S1**).

Several treatments induced alternative activation-associated genes, including *Retnla*, *Arg1*, *Ccl17*, *Ccl22*, and *Chil3* (**Figure 2A**; **Supplementary Table S1**) (39, 41). These genes were strongly expressed in IL-4-associated conditions and were also induced by GM-CSF-mediated differentiation. The concurrent induction of the inflammatory cytokine *Il1b*, the cytokine-response regulator *Socs3 (*42*)*, and alternative activation-associated genes in the IL-4 + IL-13 + IL-33 condition, together with induction of the immunoregulatory cytokine *Il10*, *Socs3*, and alternative activation-associated markers in the IL-4 + IL-13 + IL-6 condition, supports mixed or hybrid transcriptional programs rather than a strict separation between classical inflammatory and alternative activation-associated phenotypes (**Figure 2A**; **Supplementary Table S1**) (43). Consistent with these distinct transcriptional programs, macrophages treated with IL-4 + IL-13 + IL-6 or IL-4 + IL-13 + IL-33 displayed greater cell spreading and elongation than cells treated with IL-4 + IL-13 alone (**Figure 2C**). Notably, although these combinatorial conditions induced selected inflammatory markers, they did not significantly increase nitrite production, unlike IFNγ-treated macrophages (**Figure 2B**).

### 3.3 Divergent pro-inflammatory macrophage programs induced by GM-CSF, IL-1β and IFNγ

To characterize the transcriptional program generated by primary GM-CSF differentiation, we analyzed the Gmcsf_alone condition. Analysis of cytokine transcript profiles showed that Gmcsf_alone strongly induced a distinct inflammatory program characterized by significant upregulation of *Il1b*, *Ccl3*, *Ccl4*, *Ccl5*, *Cx3cl1*, *Cxcl3*, *Csf1*, and *Osm*, consistent with a highly activated, pro-inflammatory state capable of recruiting and activating additional immune cells (**Figure 3A**; **Supplementary Table S1**) (39, 44). Cytokine receptor genes including *Il1r2*, *Il2ra*, *Il13ra1*, *Il21r* were also strongly enriched in the Gmcsf_alone group (**Supplementary Figure S1A**). In addition, Gmcsf_alone upregulated a broad set of fibrosis and tissue-remodeling genes (*Fn1*, *Vcan*, *Mmp12*, *Mmp13*, *Mmp14*, *Timp1*, *Spp1*, *Pdgfa*, *Vim* etc.; **Supplementary Figure S1B**; **Supplementary Table S1**). Gene ontology analysis of the Gmcsf_alone group revealed enrichment for *wound healing*, *response to hypoxia, regulation of inflammatory response, response to molecules of bacterial origin, and leukocyte proliferation/migration* while genes associated with *response to virus* and *cell division* were downregulated (**Figure 4**; **Supplementary Figure S1C**). Although several genes highly activated by Gmcsf treatment, such as *Vcan*, *Pgf*, *Hbegf*, *Mmp8*, and *Mmp13*, were also significantly upregulated in the Mcsf.Gmcsf group, these macrophages exhibited a much milder inflammatory and fibrotic profile compared to the Gmcsf_alone group (**Supplementary Table S1**).

**Figure 3:**
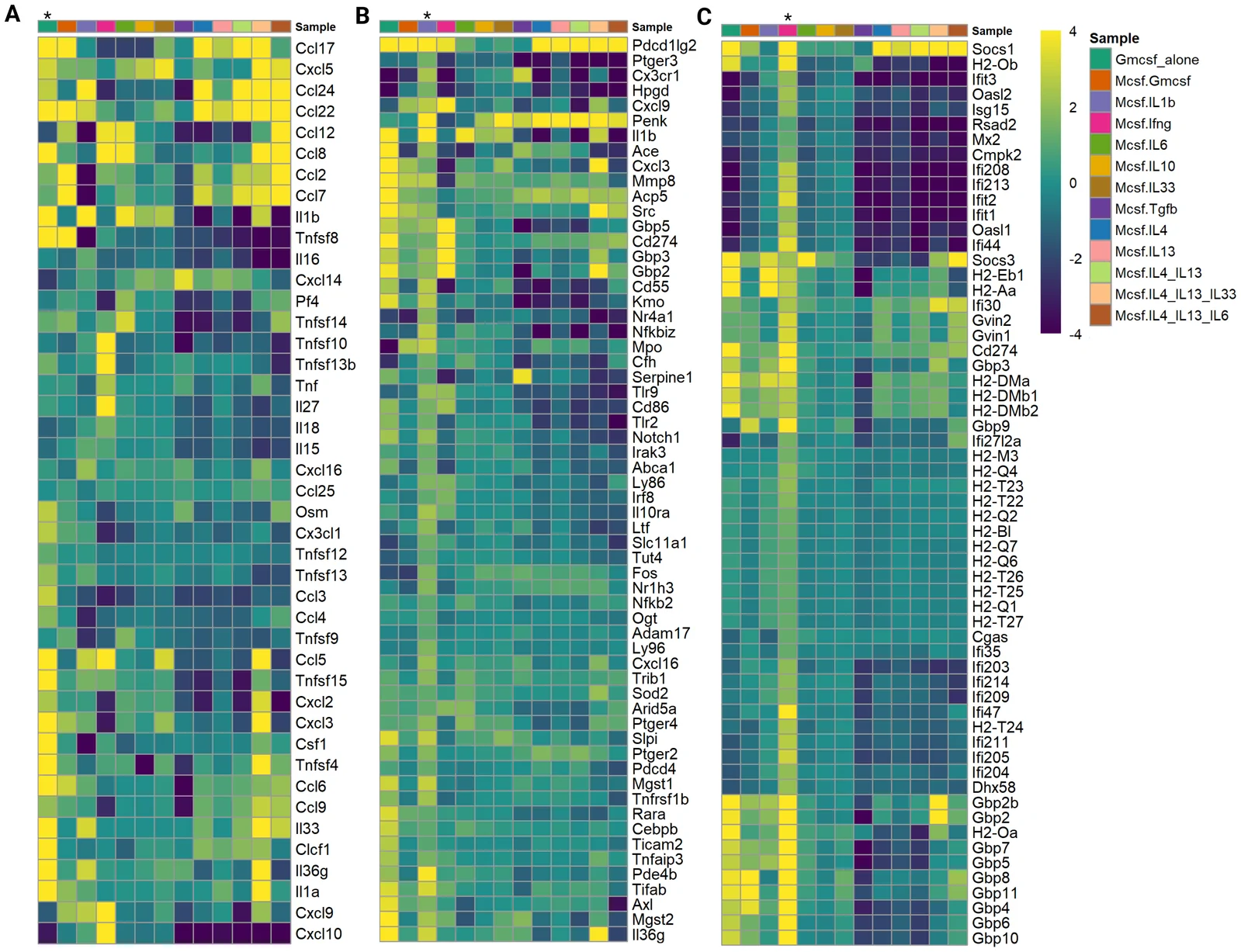
Inflammatory gene signatures in polarized macrophages. **(A)** Heatmap of cytokine- and chemokine-encoding transcripts across cytokine-polarized macrophages. Gmcsf_alone strongly induced a distinct inflammatory program (Gmcsf_alone group is indicated by *). **(B)** Heatmap depicting genes associated with response to molecules of bacterial origin activated by IL-1β (Mcsf.IL1b group is indicated by *). **(C)** Heatmap showing interferon genes and antigen presentation genes activated by IFNγ (Mcsf.Ifng group is indicated by *).

**Figure 4:**
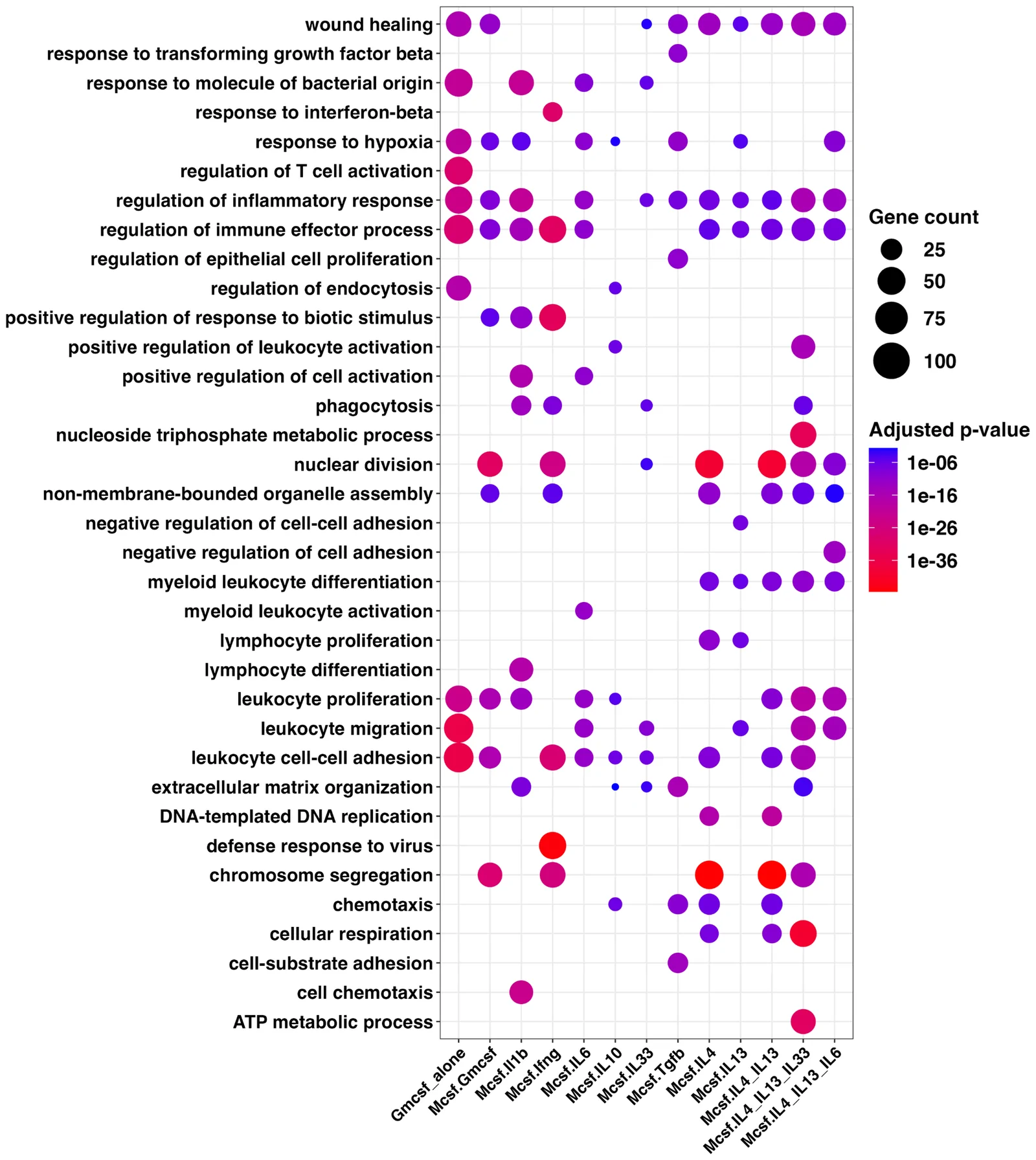
Cytokine-specific enrichment of biological processes in macrophages. Dot Plot showing the top five enriched biological processes among upregulated genes for each treatment condition. Dot size reflects the number of genes in a given process, while color intensity indicates the adjusted p-value.

IL-1β treatment (Mcsf.IL1b) significantly upregulated genes associated with a bacterial-response program, including *Tlr2*, *Tlr9*, *Irf8*, *Nfkb2*, *Il1b*, *Gbp2*, *Gbp3*, and *Gbp5* (**Figures 3B**, **4**; **Supplementary Table S1**) consistent with the established role of IL-1-family signaling in innate inflammation and antimicrobial host responses (24, 25). In contrast, IFNγ (Mcsf.Ifng) specifically induced interferon-stimulated genes and antigen-presentation machinery (*Isg15*, *Oasl2*, *Mx2*, and several H2 family genes) reflecting a classical antiviral, M1-like response (**Figures 3C**, **4**; **Supplementary Table S1**). This underscores how specific cytokine cues direct macrophages toward either antibacterial or antiviral effector states, reflecting their adaptability in host defense.

### 3.4 Alternative (M2) macrophage programs promote tissue repair and anti-inflammatory responses

To better understand alternative or M2 macrophage programs, we first examined IL-10-polarized macrophages, which are generally classified as an anti-inflammatory or immunoregulatory subtype. Principal component analysis (PCA) revealed that macrophages stimulated with IL-10 or IL-33 clustered closer to the control group than other M2 conditions, such as TGF-β1, IL-4, IL-13, and their combinations (**Figure 5A**), consistent with their relatively limited impact on global transcriptional changes (**Figure 1B**). The IL-10-responsive gene heatmap (**Figure 5B**) provided additional insight into these transcriptional programs. A substantial proportion of IL-10-regulated genes displayed concordant changes across multiple cytokine treatments, with IL-33 producing one of the most closely aligned transcriptional signatures to IL-10 (**Figures 1C**, **5B**). Notably, the serine protease inhibitor *Serpina3h* and the serine peptidase *Klk1b1*, along with *Fbp1*, *Il1rl1*, and *Itgb3*, were significantly downregulated in both IL-10- and IL-33-treated macrophages, as well as in the IL-6 group, and, with the exception of *Serpina3h*, in the TGF-β1 group, but were upregulated under several other conditions (**Figure 5B**; **Supplementary Table S1**). Conversely, IL-10 and IL-33 significantly induced genes such as *Penk*, *Mmp14* and *Fabp7*, which were also upregulated in several M2-associated macrophage groups. We also identified genes preferentially modulated by IL-33 but not by IL-10, including *Il2ra*, *Il31ra*, *Ccl5*, and *Mmp9* (**Figure 5C**; **Supplementary Table S1**). Together, these findings emphasize the context-dependent nature of macrophage gene regulation and show that IL-10 and IL-33 elicited comparatively limited transcriptional responses relative to cytokines associated with stronger inflammatory or tissue-remodeling programs.

**Figure 5:**
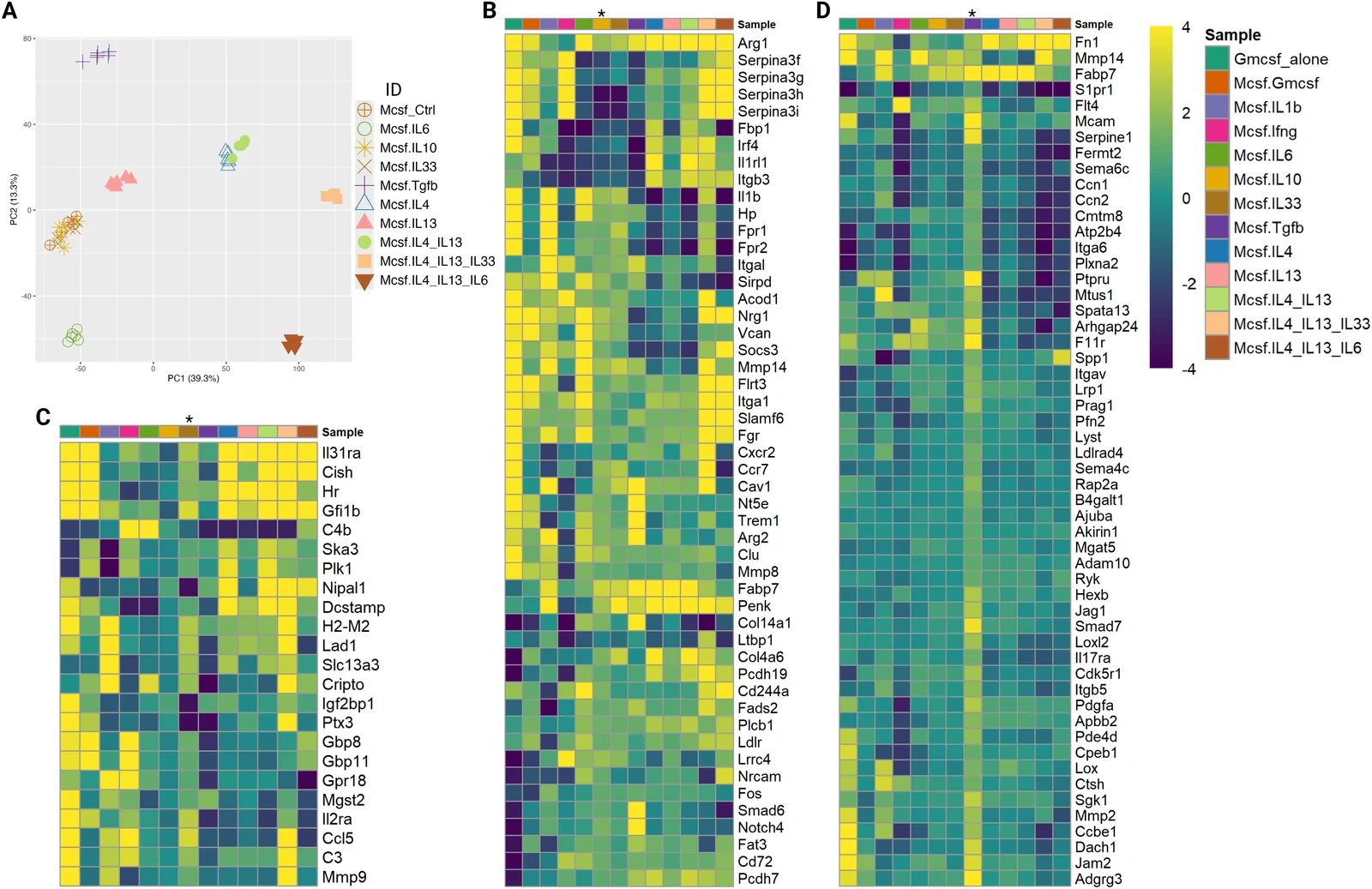
Gene-expression profiling reveals transcriptional diversity among M2-polarized macrophages. **(A)** PCA plot of global transcriptional profiles. Macrophages treated with TGF-β1 form a distinct cluster separated from all other conditions. IL-4 and IL-4+IL-13 treatments cluster closely together, whereas IL-4+IL-13+IL-33 and IL-4+IL-13+IL-6 form separate clusters. IL-33 and IL-10-treated macrophages cluster near the control group, indicating minimal transcriptional shifts. **(B)** Heatmap of a subset of IL-10-responsive genes identified in this study. IL-10 group is highlighted with *. **(C)** Heatmap of genes modulated by IL-33 but not by IL-10. IL-33 group is highlighted with *. **(D)** Heatmap of a subset of genes regulated by TGF-β1. TGF-β1 group is highlighted by *.

Treatment of macrophages with IL-6 (Mcsf.IL6) resulted in activation of genes associated with biological processes including *responses to bacterial molecules*, *inflammatory signaling*, and *myeloid leukocyte activation*, indicating a more proinflammatory phenotype (**Figure 4**). These macrophages significantly induced inflammatory genes included *Il1b*, *Ptgs2*, *Cxcl2* and *Nfkb2* (**Supplementary Figure S2A**; **Supplementary Table S1**) (39, 43). Interestingly, IL-6 also significantly induced the alternative activation-associated genes *Retnla* and *Arg1*, suggesting that IL-6 promotes a mixed or hybrid transcriptional state rather than a strictly M1- or M2-like phenotype (**Figure 2A**; **Supplementary Table S1**) (39).

Next, we examined macrophage programs induced by TGF-β1, which showed a noticeable degree of similarity to the IL-10 transcriptional profile (**Figure 1C**). TGF-β1 stimulation led to robust upregulation of genes associated with *extracellular matrix organization*, *chemotaxis*, *response to hypoxia*, *cell-substrate adhesion*, and *wound healing* (**Figure 4**). Migratory and chemotactic genes significantly induced by TGF-β1 included *Ccn1*, *Ccn2*, *Il17ra*, *Jag1*, *Mcam*, and *Pdgfa* (**Figure 5D**; **Supplementary Table S1**). Additionally, TGF-β1 strongly upregulated tissue-remodeling and fibrosis-associated genes such as *Fn1*, *Spp1*, *Mmp2*, *Mmp14*, *Lox*, *Loxl2*, and *Serpine1* (**Figure 5D**; **Supplementary Table S1**), highlighting its central role in promoting matrix remodeling. These findings underscore the dual role of TGF-β1 in facilitating tissue repair but also raise the possibility of pathological remodeling and fibrosis when its signaling is dysregulated.

Finally, we examined macrophage responses to IL-4, IL-13, and their combinations. Consistent with correlation analysis (**Figure 1C**), PCA analysis (**Figure 5A**) showed that macrophages stimulated with IL-4 or IL-4+IL-13 exhibited highly similar transcriptional profiles. However, adding IL-33 or IL-6 to the IL-4+IL-13 combination produced distinct shifts in the PCA space, indicating more nuanced modulation of gene-expression programs.

Treatments containing IL-4 alone or in combination (IL-4+IL-13, IL-4+IL-13+IL-33, IL-4+IL-13+IL-6) consistently show enrichment for processes including *wound healing*, *myeloid leukocyte differentiation*, and *cell division-associated pathways* and downregulation of *viral response pathways* (**Figure 4**; **Supplementary Figure S1C**). *ATP metabolic process* and *cellular respiration* were strongly enriched in the IL-4+IL-13+IL-33 group, suggesting the emergence of a distinct metabolic state (**Figure 4**). Upregulation of wound healing and tissue repair-associated genes (*F3, Hbegf, Klf4, Vwf, Pros1, Htr2a, Plau, Plaur, Mmp12, Mmp13, Timp1*, etc.) supports the premise that IL-4-driven programs promote reparative macrophage phenotypes (**Figure 6A**; **Supplementary Table S1**). The addition of IL-6 to IL-4+IL-13 further amplified the expression of several of these genes, such as *Hbegf, Klf4*, and *Timp1*, consistent with a stronger wound-healing-associated transcriptional program (**Supplementary Table S1**). IL-4 and IL-4-combination treatments also contributed to pronounced upregulation of angiogenesis-associated genes, with key regulators such as *Epha1, Sphk1, Hhip, Ntrk1, Flt1, Itgb8* showing strong expression (**Figure 6B**; **Supplementary Table S1**).

**Figure 6:**
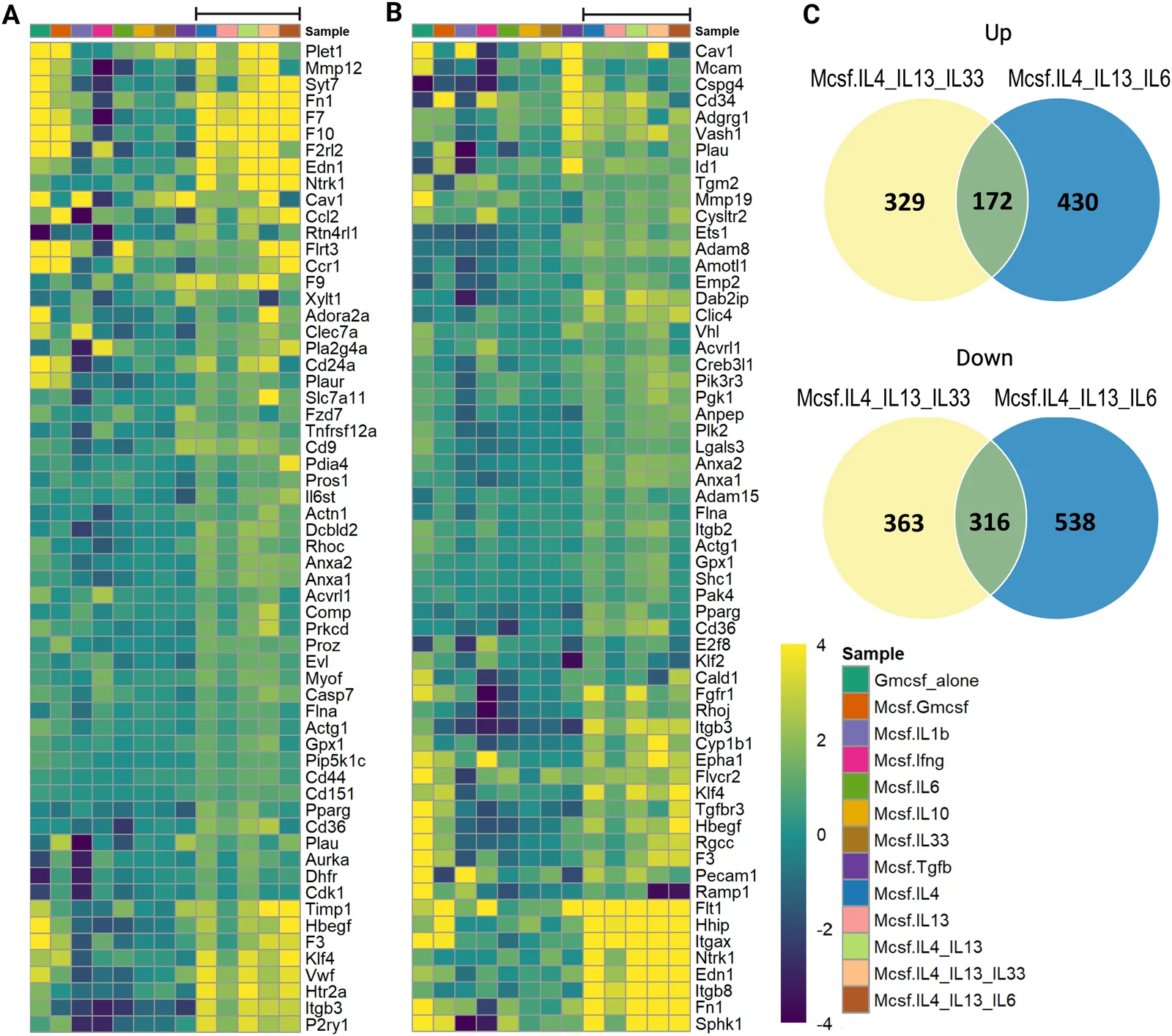
Gene-expression profiling reveals transcriptional diversity among macrophages stimulated with IL-4 and its combinations. **(A)** Heatmap of a subset of genes associated with wound healing. IL-4 and its combinations (highlighted) show enhanced expression of wound healing genes. **(B)** Heatmap of a subset of genes associated with angiogenesis. IL-4 and IL-4-combination treatments (highlighted) contributed to pronounced upregulation of angiogenesis-associated genes. **(C)** Venn diagram showing the overlap between genes modulated by the addition of IL-6 or IL-33 to IL-4+IL-13 relative to IL-4+IL-13 alone.

To further define how IL-6 and IL-33 modify IL-4 + IL-13-polarized macrophages, we compared the IL-4 + IL-13 + IL-6 and IL-4 + IL-13 + IL-33 conditions with the IL-4 + IL-13 backbone (**Figure 6C**; **Supplementary Table S2**). A total of 172 upregulated and 316 downregulated genes were shared between the two combinations; however, most differentially expressed genes were unique to either condition, indicating that IL-6 and IL-33 produce distinct transcriptional responses on an IL-4/IL-13-associated background (**Figure 6C**; **Supplementary Table S2**).

Both combinations were enriched for tissue-remodeling and interferon-mediated signaling programs (**Supplementary Figure S2B**). Shared remodeling-associated genes included *Mmp14*, *Timp1*, *Tgm1*, *Vcan*, and *Tgfbi*, whereas shared interferon-associated genes included *Irf7*, *Usp18*, *Jak2*, and *Sting1* (**Supplementary Table S2**; **Supplementary Figure S2B**). Both conditions also induced inflammatory and cytokine-response genes, including *Nos2*, *Cxcl5*, *Socs3*, and *Casp4* (**Supplementary Table S2**). Thus, IL-6 and IL-33 each added inflammatory, interferon-associated, and remodeling features to the IL-4 + IL-13 backbone (**Supplementary Table S2**; **Supplementary Figure S2B**).

In addition, the IL-4 + IL-13 + IL-6 condition showed a prominent immunoregulatory and alternative activation-associated component, with increased expression of *Il10*, *Fgl2*, *Cd274*, *Fcgr2b*, *Mrc1, Cd163* and *Il4ra* (**Supplementary Table S2**). It also increased expression of *Vegfd*, *Spp1*, and *Pdpn*, consistent with vascular and tissue-remodeling features. *Socs3*, a negative-feedback regulator of cytokine signaling (42), was induced in both combinations but showed greater relative induction in the IL-6 condition, consistent with active cytokine signaling and feedback regulation. The IL-6-containing condition also showed enrichment of amino acid metabolic pathways, including genes involved in arginine/nitric oxide metabolism (*Ass1, Nos2, Arg1*, and *Arg2*), serine and asparagine biosynthesis (*Phgdh, Psph*, and *Asns*), and additional amino acid metabolic pathways (*Kmo, Gpt2, Odc1, Aldh18a1, Tha1, Adhfe1*, and *Bcat1*) (**Supplementary Table S2**). In contrast, the IL-4 + IL-13 + IL-33 condition was distinguished by strong induction of inflammatory and chemoattractant genes, including *Cxcl2*, *Cxcl3*, *Ccl5*, *Cxcl16*, *Il1b*, *Il1a*, *Il36g*, *Tnf*, and *Tnfsf4*. IL-33 addition also increased *Ccl17* and *Ccl22*, indicating concurrent induction of chemokines associated with type 2 and regulatory immune responses (**Supplementary Table S2**). In addition, the IL-33-containing condition showed enrichment of a sterol-biosynthetic program, supported by increased expression of *Msmo1*, *Mvk*, *Mvd*, *Fdft1*, *Nsdhl*, *Lss*, *Sqle*, *Sc5d*, *Cyp51*, and *Dhcr24* (**Supplementary Table S2**; **Supplementary Figure S2B**), although a few of these genes were also increased following IL-6 addition (**Supplementary Table S2**).

Together, these findings show that IL-6 and IL-33 do not simply amplify the IL-4 + IL-13 program. Instead, IL-6 adds immunoregulatory and alternative activation-associated programs with remodeling features, whereas IL-33 combines tissue remodeling with a broader chemotactic, inflammatory, and sterol metabolic response.

Next, we used decoupleR to infer transcription factor activities associated with alternative activation-associated transcriptional programs, relative to naive macrophage controls. We identified condition-specific regulon activity across IL-4/IL-13-associated conditions and examined the corresponding predicted target genes (**Figure 7A**; **Supplementary Tables S3, S4**). IL-4-based combination treatments showed strong enrichment of Myc (c-Myc), a transcription factor implicated in macrophage proliferation, metabolic reprogramming, and alternative activation (45, 46). Predicted Myc regulon in these macrophages included a large number of genes associated with cell differentiation and proliferation including *Cdk1*, *Ccnb1*, *Ccnb2*, *Ccnd1* and *Cdc20* (**Supplementary Table S4**). Klf4 was also enriched in IL-4 and its combination groups (**Supplementary Table S3**), and both *Klf4* and *Myc* were transcriptionally upregulated in these conditions (**Figure 7B**). Although *Irf4* was transcriptionally upregulated across all IL-4 combination treatments, significant enrichment for Irf4 activity was predicted exclusively in the IL-33 and IL-4+IL-13+IL-33 groups (**Figure 7B**; **Supplementary Table S3**).

**Figure 7:**
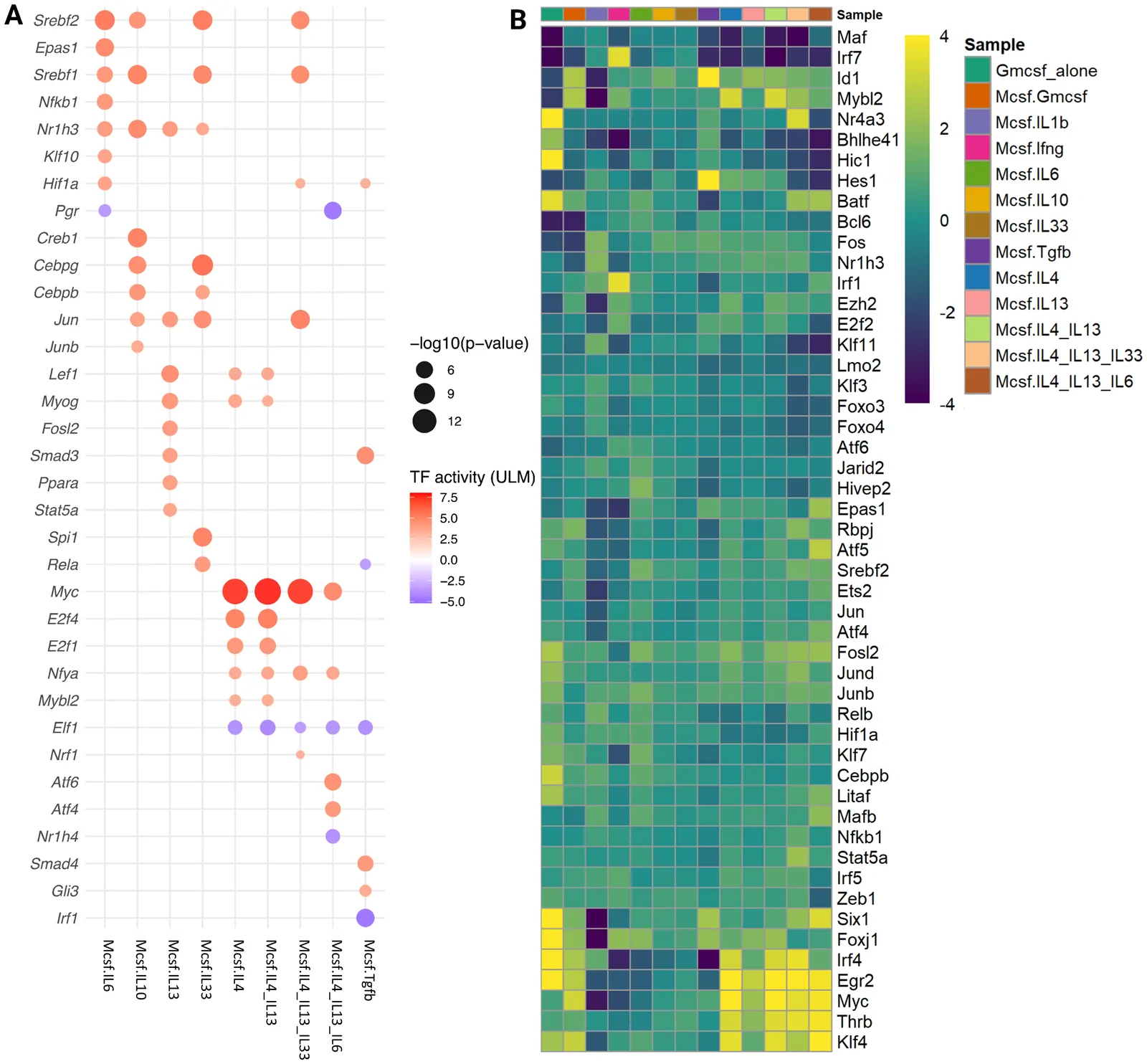
Inferred transcriptional-regulatory profiles across alternative activation-associated macrophage conditions. **(A)** Dot plot displays the top transcription factors (TFs) per condition. Each dot represents a TF-condition pair, with dot size proportional to −log_10_(p-value), indicating statistical significance. Dot color reflects TF activity score. **(B)** Heatmap summarizing differential gene expression of selected TFs across conditions.

Srebf1 and Srebf2, transcription factors that play key roles in regulating lipid and cholesterol metabolism, showed enriched regulon activity in IL-6-, IL-10-, and IL-33-treated single and combination conditions (**Figure 7A**) (47). Their predicted targets included genes associated with fatty-acid and lipid metabolism, such as *Fasn* and *Pparg*, as well as cholesterol biosynthesis and uptake genes, including *Hmgcs1*, *Hmgcr*, *Fdft1*, *Sqle*, *Cyp51*, *Dhcr24*, and *Ldlr* (**Supplementary Table S4**). Many of these genes were also transcriptionally upregulated in the IL-4 + IL-13 + IL-33 condition relative to the IL-4 + IL-13 backbone (**Supplementary Table S2**).

IL-6 stimulation alone showed strong enrichment for Nfkb1, a central mediator of inflammatory gene induction, and Epas1 (Hif2a), a hypoxia-responsive transcription factor implicated in metabolic and inflammatory reprogramming of macrophages (**Figure 7A**; **Supplementary Table S3**) (48–50). In contrast, IL-6 combined with IL-4 and IL-13 showed strong enrichment of transcription factors Atf4 and Atf6 that coordinate amino-acid stress responses and ER stress-linked remodeling of macrophage function, suggesting that IL-6 engages distinct regulatory modules under inflammatory versus reparative/type-2 cytokine contexts (**Supplementary Table S3**) (51, 52). Consistent with this, both predicted Atf4 and Atf6 regulons in IL-4 + IL-13 + IL-6-stimulated macrophages included *Hspa5*, a core component of the ER stress adaptation machine (**Supplementary Table S4**) (53). Predicted Atf4 targets included genes associated with amino acid biosynthesis, metabolism, and stress adaptation, including *Asns*, *Gpt2*, *Iars1*, *Trib3*, and *Chac1* (**Supplementary Table S4**). These genes were also transcriptionally upregulated in the IL-4 + IL-13 + IL-6 condition relative to the IL-4 + IL-13 backbone (**Supplementary Table S2**). In contrast, predicted Atf6 targets included genes involved in ER protein folding and quality control, including *Hsp90b1*, *Dnajc3*, *Hyou1*, *Calr*, and *Pdia6*, together with *Derl3*, *Wfs1*, and *Manf*, consistent with ER stress-adaptation features (**Supplementary Table S4**).

TGF-β1 stimulation enriched for Smad2, Smad3 and Smad4, the central mediators of canonical TGF-β/Smad signaling, as well as Gli3, reflecting known crosstalk between TGF-β and Hedgehog pathways (**Figures 7A, B**; **Supplementary Table S3**). Id1 was both enriched and transcriptionally upregulated in the TGF-β1 group (**Figures 7A, B**; **Supplementary Table S3**). Transcription factors from the Jun/Fos family including Fos, Fosl2, Jun and Junb were enriched in IL-10 group as well as several other alternatively activated conditions, many of which were also transcriptionally upregulated, highlighting activation of AP-1-mediated pathways (**Figures 7A, B**; **Supplementary Tables S1, S3**).

Although they did not show positive predicted regulon enrichment, *Mafb, Batf*, and *Six1* transcription factor transcripts were upregulated across multiple IL-4-containing combinations (**Supplementary Table S1**, **S3****;** **Figure 7B**). Together, these findings show that the cytokine conditions were associated with distinct patterns of transcription factor activity and transcription factor expression.

### 3.5 Validation of macrophage hybrid activation states at the secretory protein level

To determine whether the transcriptionally identified hybrid and noncanonical activation states translate into functional protein expression, we analyzed the macrophage secretome using a multiplex cytokine array following 24 h of polarization (**Figure 8**). The secreted-protein profiles supported the transcriptomic finding that combinatorial cytokine cues produce overlapping inflammatory, immunoregulatory, and alternative activation-associated responses rather than discrete M1- or M2-like states.

**Figure 8:**
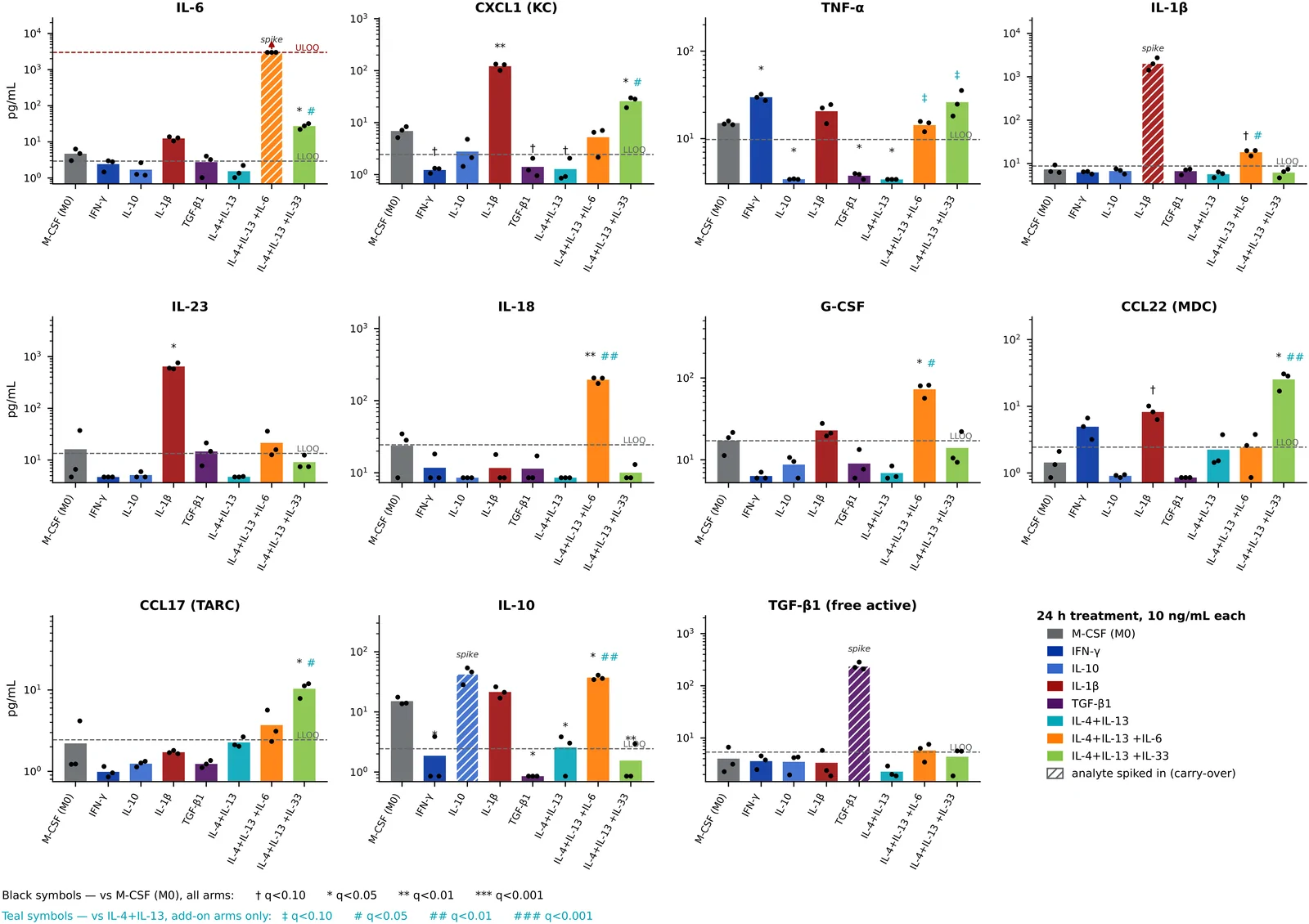
Multiplex cytokine and chemokine profiling identifies distinct secretory responses during combinatorial polarization. Mouse BMDMs were treated for 24 h with the indicated individual cytokines or selected combinations (10 ng/mL each). Secreted protein concentrations (pg/mL) were measured using a multiplex array for inflammatory cytokines and chemokines (IL-6, CXCL1, TNF-α, IL-1β, IL-18, G-CSF, and IL-23) and alternative activation-associated or regulatory factors (CCL22, CCL17, IL-10, and TGF-β1). IL-12p40 and IL-12p70 levels were measured but are not shown because no significant differences were detected among conditions. Bars represent the mean of three replicate culture wells from a single BMDM preparation; dots represent individual culture wells, each measured in duplicate technical assay reads. Dashed horizontal lines indicate the upper (ULOQ) or lower (LLOQ) limit of quantitation. Hatched bars labeled “spike” indicate exogenous cytokine carry-over from the treatment medium rather than endogenous cellular secretion. Statistical significance was evaluated using Welch’s t-test with Benjamini–Hochberg false-discovery-rate correction. Symbols above bars indicate comparisons with the M-CSF (M0) baseline: †, q < 0.10; *q < 0.05; **q < 0.01; ***q < 0.001. Teal symbols above combination-treatment bars indicate comparisons with the IL-4 + IL-13 base treatment: ^‡^q < 0.10; ^#^q < 0.05; ^##^q < 0.01; ^###^q < 0.001.

TNF-α secretion was significantly increased in IFNγ-treated macrophages, with a smaller, non-significant increase in IL-1β-treated macrophages, whereas IL-1β, but not IFNγ, strongly induced secretion of the neutrophil-recruiting chemokine CXCL1 (KC) (**Figure 8**). Consistent with the transcriptional data (**Supplementary Table S2**), addition of IL-33 to the IL-4 + IL-13 backbone increased secretion of CCL17 (TARC) and CCL22 (MDC). In contrast, addition of IL-6 to IL-4 + IL-13 uniquely increased secretion of the immunoregulatory cytokine IL-10, consistent with increased Il10 expression in this condition (**Figure 8**; **Supplementary Table S2**). Importantly, despite the presence of the anti-inflammatory IL-4+IL-13 backbone, the addition of either IL-6 or IL-33 significantly elevated the release of TNF-α, CXCL1 (KC), IL-18 or G-CSF (**Figure 8**). Together, these results show that combinatorial cytokine signaling generates distinct mixed secretory programs and reinforces the context-dependent nature of macrophage activation.

## 4 Discussion

Consistent with the current understanding that macrophage activation exists along a continuum rather than a strict M1/M2 dichotomy, our study provides a systematic transcriptomic comparison of macrophage responses across a broad panel of inflammatory, regulatory, and reparative cytokines. By performing a controlled, side-by-side comparison in a defined *in vitro* system, we identify distinct and overlapping transcriptional programs induced by individual cytokines and cytokine combinations, enabling clearer attribution of cytokine-specific responses and functional states.

Consistent with previous studies, IL1-β and IFNγ induced pro-inflammatory macrophage programs (24–26). However, IL1-β preferentially induced gene signatures associated with bacterial response pathways, whereas IFNγ primarily promoted antiviral and antigen-presentation programs. These findings highlight the functional specialization of macrophages in response to distinct inflammatory cues.

GM-CSF-differentiated macrophages displayed a distinct transcriptional profile characterized by concurrent expression of inflammatory, immunoregulatory, and tissue-remodeling-associated genes. GM-CSF-driven cultures can be heterogeneous and may include macrophage- and dendritic cell-like populations, which could contribute to this broadened signature (54, 55). While GM-CSF is widely recognized as a driver of inflammatory monocyte and macrophage differentiation, particularly in autoimmune and chronic inflammatory settings (56, 57), our data identified tissue remodeling-associated programs alongside inflammatory programs. In contrast, M-CSF-differentiated macrophages subsequently exposed to GM-CSF showed a more modest transcriptional shift, suggesting that prior differentiation conditions influence subsequent responsiveness to GM-CSF.

IL-4-based treatments, particularly in combination with IL-13, induced transcriptional programs with alternative activation-associated and tissue-remodeling features. These findings are consistent with prior studies showing that IL-4 and IL-13 signal through STAT6 to promote alternative macrophage activation in both human and murine systems (13, 22). Consistent with this literature, our study (**Figures 1C**, **5A**) showed that IL-4 and IL-13 induced highly similar transcriptional states in BMDMs.

Addition of IL-6 or IL-33 to the IL-4 + IL-13 backbone further modified the associated transcriptional responses. Direct comparison with the IL-4 + IL-13 condition showed that neither cytokine uniformly amplified the IL-4/IL-13-associated program. Both combinations shared inflammatory, interferon-associated, and tissue-remodeling features; however, IL-6 addition was associated with increased expression of immunoregulatory and alternative activation-associated genes, including *Il10*, *Fgl2*, *Pdcd1lg2*, *Fcgr2b*, *Mrc1*, and *Il4ra*, as well as vascular and remodeling-associated genes such as *Vegfd*, *Spp1*, and *Pdpn*. IL-6 addition was also associated with increased expression of genes involved in amino-acid metabolism and stress adaptation, including *Asns*, *Gpt2*, and *Iars1*, together with *Trib3* and *Chac1*, consistent with a distinct metabolic and stress-response component. In contrast, IL-33 addition was characterized by stronger induction of chemotactic and inflammatory genes, including *Cxcl2*, *Cxcl3*, *Ccl5*, *Il1a*, *Il1b*, and *Tnf*, together with a sterol-biosynthetic program. Representative imaging further showed visibly distinct cell-shape and spreading patterns in the IL-4 + IL-13 + IL-6 and IL-4 + IL-13 + IL-33 conditions relative to the IL-4 + IL-13 backbone (**Figure 2C**). Although descriptive, these morphological differences were consistent with the distinct transcriptional programs associated with IL-6 and IL-33 addition.

The cytokine profiles further support the conclusion that IL-6 and IL-33 modify, rather than simply intensify, the IL-4 + IL-13-associated macrophage program. IL-33 addition favored secretion of the type 2-associated chemokines CCL17 and CCL22, whereas IL-6 addition was associated with increased IL-10 secretion. At the same time, detection of TNF-α, CXCL1, and IL-1β indicates that these cells retain inflammatory secretory capacity. Thus, the combined cytokine environments generated distinct outputs incorporating alternative, regulatory, and inflammatory features simultaneously.

The inferred regulatory profiles suggest that IL-4/IL-13-associated activation comprises multiple, context-dependent programs rather than a single transcriptional state. IL-4-containing conditions showed predicted MYC regulon activity together with increased *Myc* expression, consistent with a shared regulatory component across these treatments. Addition of IL-6 or IL-33 to the IL-4 + IL-13 backbone was associated with distinct predicted target-gene profiles: the IL-6 combination showed ATF4/ATF6-associated changes consistent with amino acid metabolic and stress-response features (52, 58), whereas IL-33 addition was associated with SREBF1/SREBF2-related target genes, consistent with the accompanying sterol-biosynthetic program (59). Increased expression of *Mafb* (60), *Six1* (61) and *Klf4* (62, 63) across alternative activation-associated conditions further identifies candidate regulators that may contribute to diversification of these states. These analyses generate testable regulatory hypotheses but do not establish direct causal roles for these factors in macrophage activation or tissue repair.

A principal strength of this study is the controlled, side-by-side comparison of macrophage responses to individual and combinatorial cytokine environments from a common M-CSF-derived baseline. This design enabled direct assessment of how IL-6 and IL-33 modify the IL-4 + IL-13-associated transcriptional program, while complementary cytokine-secretion, nitrite, and morphology assays supported distinct functional features of these states. However, the study is limited by its reliance on controlled *in vitro* polarization systems and the absence of *in vivo* validation. Although the experiments presented here were conducted exclusively in murine macrophages, some mechanistic studies cited throughout the manuscript were performed in human macrophages. These findings were interpreted in the context of evidence from both species, as core macrophage activation pathways are often conserved, although species-specific differences in transcriptional responses have been reported. Accordingly, the extent to which the cytokine-driven programs identified here are conserved in human macrophages remains to be determined. Future studies should define the functional consequences of these macrophage states *in vivo*, particularly during inflammation resolution, wound healing, fibrosis, and tissue repair.

## 5 Conclusion

Our study provides a controlled, systematic comparison of macrophage transcriptional responses to individual and combinatorial cytokine cues. IFNγ and IL-1β elicited distinct inflammatory programs, whereas IL-4- and IL-13-containing conditions induced alternative programs characterized by tissue-repair and matrix-remodeling features. Addition of IL-6 or IL-33 to the IL-4 + IL-13 backbone further modified these states, generating distinct mixed transcriptional and secretory profiles that incorporated inflammatory, immunoregulatory, metabolic, and alternative activation features. Building on the established recognition that macrophage activation extends beyond the traditional M1/M2 framework, these results identify cytokine-responsive pathways and candidate mixed activation programs for future investigation in inflammation resolution, fibrosis, and tissue repair. Ultimately, this comprehensive functional atlas serves as a valuable resource for deciphering the mechanisms governing macrophage plasticity and immune regulation.

## Data Availability

The datasets presented in this study can be found in online repositories. The names of the repository/repositories and accession number(s) can be found in the article/supplementary material.
