# Supplementary material for: A transcriptomic atlas of macrophage polarization induced by inflammatory and reparative cytokines reveals cytokine-specific and synergistic polarization programs
Source: Front Immunol. 2026 Oct 7;17:1815547. doi: 10.3389/fimmu.2026.1815547 (PMC13683169; doi:10.3389/fimmu.2026.1815547)
Supplement: Supplementary file 1 [file DataSheet1.pdf]

Supplementary Figures

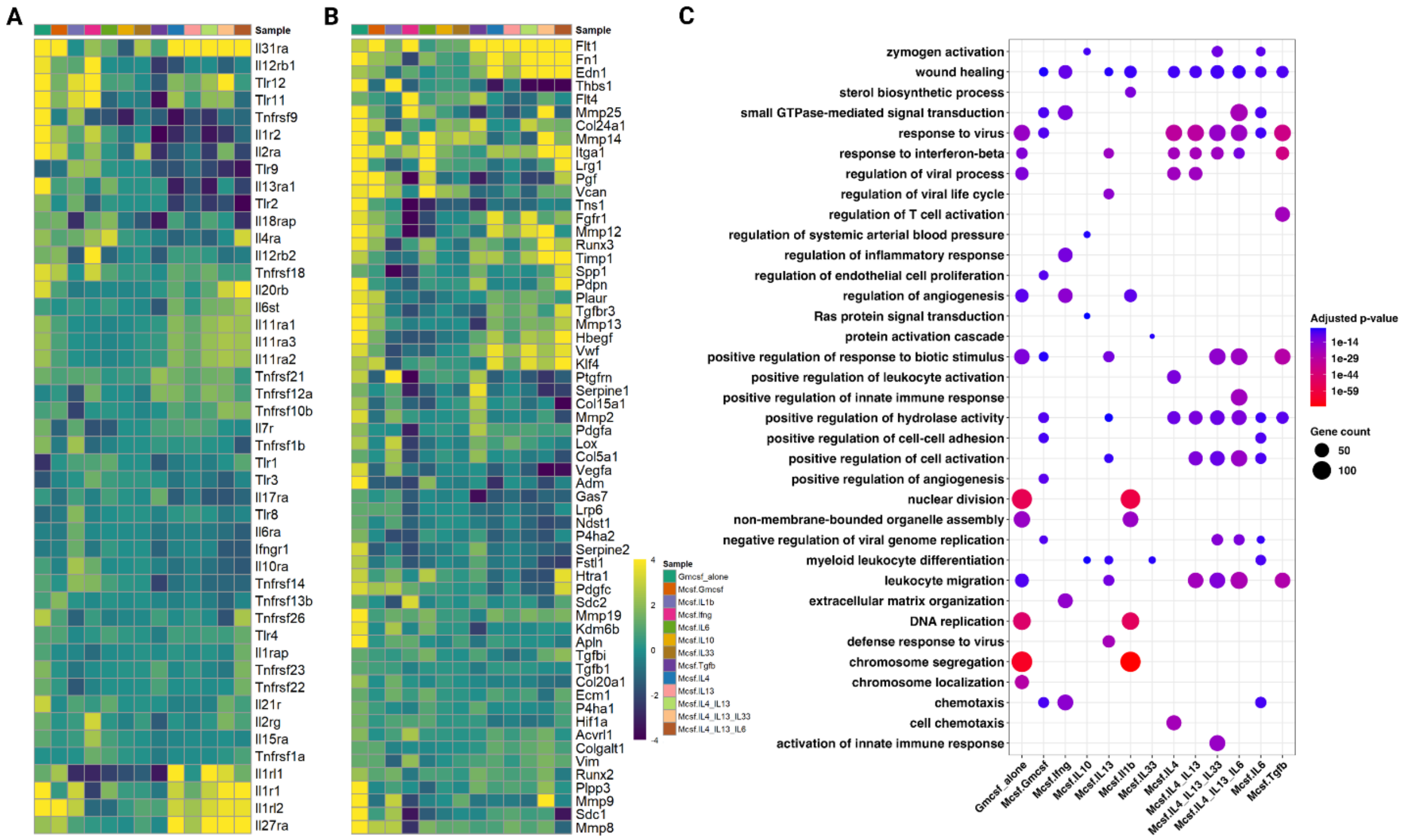

Supplementary Figure 1. Cytokine Receptor Expression, Gmcsf-Induced Remodeling Responses, and Functional Pathways Across Macrophage Polarization States (A) Heatmap displaying the expression profiles of key cytokine receptors across differentiated and polarized macrophage groups. **(B)** Heatmap depicting the tissue remodeling and fibrosis genes upregulated in Gmcsf-alone group. **(C)** Dot plot illustrating significantly enriched GO terms among downregulated genes for each experimental group. Circle size represents the number of genes, and color intensity denotes adjusted p-values.

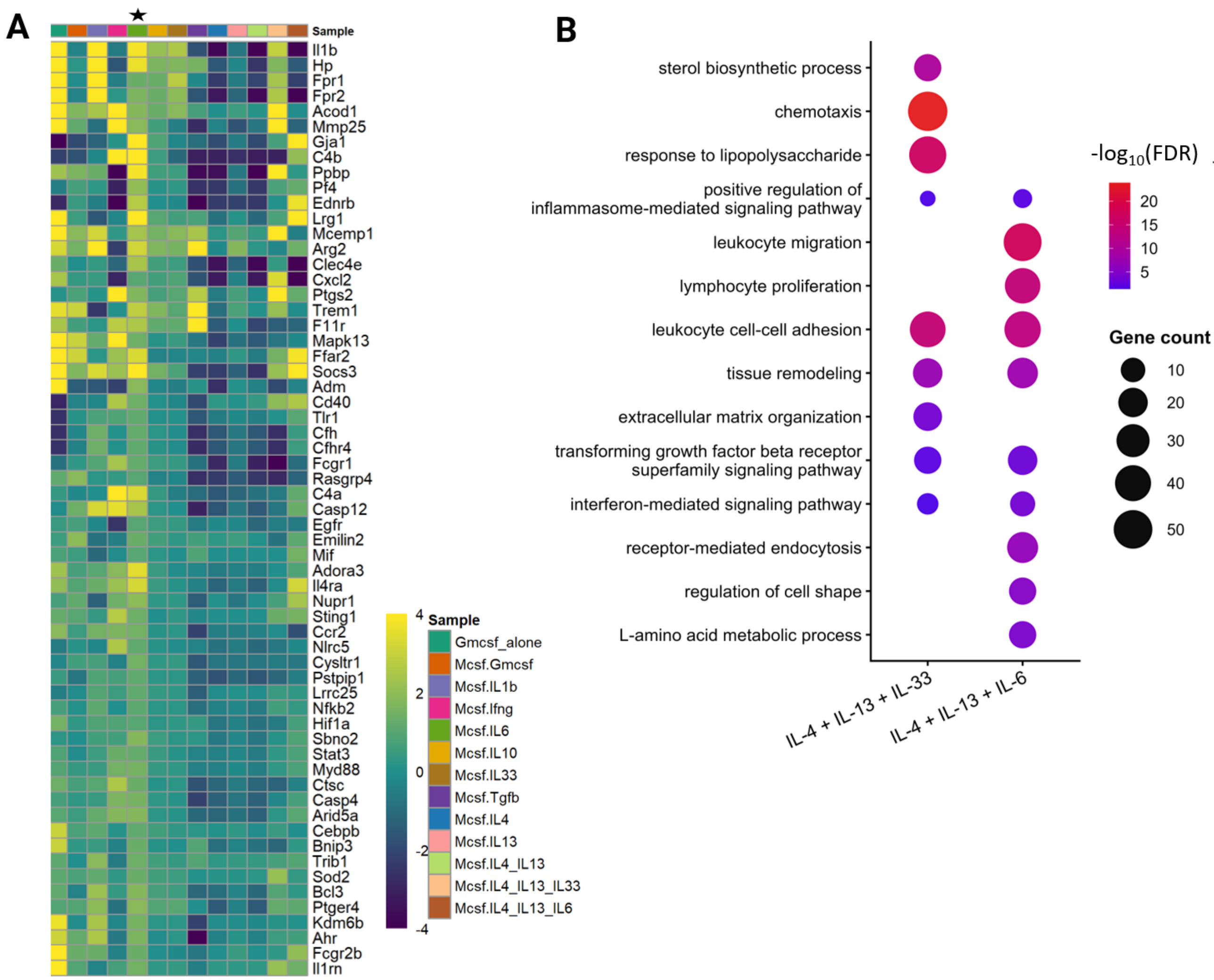

Supplementary Figure 2. IL33 and IL6-Driven Transcriptional and Functional Signatures in Polarized Macrophages (A) Heatmap showing the expression levels of IL6-responsive genes (indicated by *). **(B)** Gene Ontology biological-process enrichment among genes upregulated by addition of IL-33 or IL-6 to the IL-4 + IL-13 backbone. Terms were selected to represent nonredundant, biologically interpretable programs. Dot size indicates the number of genes associated with each term, and color indicates the FDR-adjusted enrichment significance.
